# Characterization of Left Atrial Appendage Closure Device Protrusion and Implications for Transcatheter Mitral Valve Replacement

**DOI:** 10.1016/j.jacadv.2023.100629

**Published:** 2023-09-28

**Authors:** Lauren S. Ranard, Kenneth Guber, Jay Leb, Mark Lebehn, Vratika Agarwal, Rebecca T. Hahn, Vivian Ng, Martin B. Leon, Robert Sommer, Torsten P. Vahl

Several transcatheter mitral valve replacement (TMVR) therapies are under investigation for the treatment of mitral regurgitation. A common comorbid condition is atrial fibrillation, and left atrial appendage closure (LAAC) is often performed in patients with an elevated bleeding risk.^1^ TMVR devices have different design features, but one common requirement for many of these devices is an appropriate landing zone for the atrial component of the prosthesis.^2^ Therefore, protrusion of the LAAC device into the left atrium (LA) has the potential to interact with a TMVR device. The aim of this study was to characterize LAAC device protrusion into the LA and evaluate the possible effect of this on future TMVR therapy.

**Figure 1:**
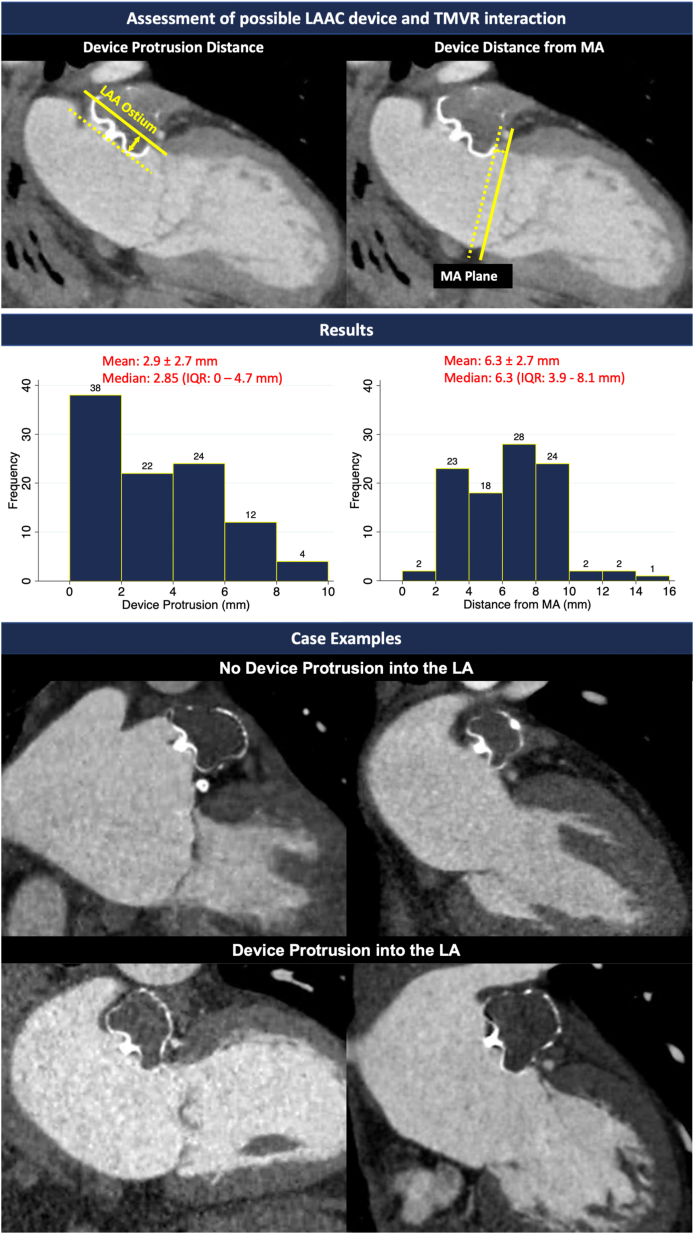
**Computed Tomography Assessment of WATCHMAN FLX Device Protrusion From Left Atrial Appendage With Case Examples** LA = left atrium; LAA = left atrial appendage; LAAC = left atrial appendage closure; MA = mitral annulus; TMVR = transcatheter mitral valve replacement.

This was a single-center, retrospective study including 100 atrial fibrillation patients who underwent LAAC with the WATCHMAN FLX device and completed a 45-day postimplant cardiac computed tomography scan. Institutional review board approval was obtained. The postimplant computed tomography was evaluated for LAAC device protrusion into the LA and distance from the mitral annulus (MA) plane (Figure 1). The authors declare that all supporting data are available within the article. Statistical analyses were performed using Stata Version 12.1 (StataCorp). All data are presented as mean ± SD.

The cohort was 75.7 ± 8.1 years of age with a CHADS-VASc of 4.5 ± 1.5 and HASBLED of 4.0 ± 1.0. The device protrusion distance was 2.9 ± 2.7 mm (range 0-8.9 mm). The distance of the LAAC device from the MA was 6.3 ± 2.7 (range 0-14.8 mm). Thirty-seven patients had no device protrusion into the LA. Analysis excluding those without protrusion demonstrated a device protrusion of 4.6 ± 1.8 mm (range 1.5-8.9 mm) and distance of the LAAC device from the MA of 5.9 ± 2.5 mm (range 2.2-14.8 mm). There was no difference in LAAC device distance from MA by mitral regurgitation severity (*P* = 0.10, Kruskal-Wallis test).

There is overlap in the populations that benefit from LAAC and mitral valve intervention. As such, there is growing interest in the combined treatment, as it is more frequently performed on surgical patients. A registry collecting data on outcomes for patients undergoing simultaneous LAAC and transcatheter mitral valve edge-to-edge repair is currently ongoing (NCT00494347).^3^ However, the need for future TMVR cannot always be foreseen at the time of LAAC. With multiple devices under investigation for TMVR, consideration of the size and placement of a LAAC device and its possible interaction with future TMVR therapy is gaining importance.

The surgical literature has described interaction between LAAC devices and surgical mitral valve bioprostheses, though the incidence of how often this problem occurs remains uncertain.^4^ The placement of a TMVR device with an atrial flange may be more challenging in patients after LAAC leading potentially to mitral paravalvular leak. However, device protrusion into the LA may be less of an issue for TMVR in patients with a larger device distance from the MA. Therefore, both of these measurements should be considered when discussing possible TMVR LAAC interaction and in the selection of TMVR devices. Another consideration is the distance from the interatrial septum to the lateral LA wall, which can be limited in some patients. Further reduction in this distance due to LAAC device protrusion may create challenges for the steering of TMVR devices with a larger bending radius. Similarly, in patients undergoing LAAC after prior TMVR, possible device interaction and secure device placement need to be taken into consideration.

Two strategies to reduce TMVR LAAC interaction are: 1) avoiding aggressive oversizing; and 2) aiming for coaxial LAAC deployment flush with the ostium. However, this is not always possible depending on the anatomy. On the other hand, overly deep implantation should be avoided, as it can increase the risk of device-related thrombosis. Patient-specific simulation with computational modeling may be helpful for determining optimal size and position.

In conclusion, LAAC device protrusion was observed in more than half of the patients in our study and therefore needs to be taken into consideration when screening patients for TMVR. Our data suggests that the MA and LAA ostium are in close proximity in a majority of patients, thus it may be beneficial for LAAC operators to avoid device protrusion into the LA whenever possible. This would then provide the greatest flexibility for future TMVR implantation, if needed. Although this paper focuses on interaction specifically with the WATCHMAN FLX device, there is a growing number of LAAC devices. Therefore, future investigations will be needed to evaluate the differential interaction of TMVR with alternative LAAC devices.
